# Epigenetic synthetic lethality approaches in cancer therapy

**DOI:** 10.1186/s13148-019-0734-x

**Published:** 2019-10-07

**Authors:** Haoshen Yang, Wei Cui, Lihui Wang

**Affiliations:** Department of Pharmacology, Shenyang Pharmaceutical University, Shenyang, 110016 People’s Republic of China

**Keywords:** Epigenetic regulation, Synthetic lethal, Cancer, SWI/SNF, Mutation

## Abstract

The onset and development of malignant tumors are closely related to epigenetic modifications, and this has become a research hotspot. In recent years, a variety of epigenetic regulators have been discovered, and corresponding small molecule inhibitors have been developed, but their efficacy in solid tumors is generally poor. With the introduction of the first synthetic lethal drug (the PARP inhibitor olaparib in ovarian cancer with *BRCA1* mutation), research into synthetic lethality has also become a hotspot. High-throughput screening with CRISPR-Cas9 and shRNA technology has revealed a large number of synthetic lethal pairs involving epigenetic-related synthetic lethal genes, such as those encoding SWI/SNF complex subunits, PRC2 complex subunits, SETD2, KMT2C, and MLL fusion proteins. In this review, we focus on epigenetic-related synthetic lethal mechanisms, including synthetic lethality between epigenetic mutations and epigenetic inhibitors, epigenetic mutations and non-epigenetic inhibitors, and oncogene mutations and epigenetic inhibitors.

## Background

Epigenetics is a term which describes heritable changes of gene expression without alteration of the DNA sequence [1]. Epigenetic modifications include DNA methylation, histone modification (methylation, acetylation, phosphorylation and ubiquitination), chromatin remodeling, and RNA methylation [2]. Epigenetics has become a hot topic in the field of malignant tumor treatment. A variety of epigenetic-related genes have been found to be associated with tumor development, progression and drug resistance [3], and small molecule inhibitors targeting the protein products of these genes have also been synthesized. However, in clinical studies, these inhibitors are only efficacious against some hematologic malignancies and are not very effective for treating most solid tumors [2].

As early as 1922, there was a report about the synthetic lethality phenomenon, when Calvin Bridges observed that simultaneous mutation of a pair of genes in a fruit fly would lead to death, but mutation of either gene alone did not significantly affect survival [4]. The same phenomenon was observed at the cellular level thereafter. Functional changes in two genes resulted in cell death, whereas survival was maintained if either gene was changed.

Loss-of-function (LOF) mutations or low expression of tumor suppressor genes are very common in cancer. Compared to gain-of-function (GOF) mutations or overexpression, mutations that cause loss of function or low expression of tumor suppressor genes are considered as “undruggable” [5]. However, screening for synthetic lethal interactions can be performed by shRNA or CRISPR-Cas9 systems, with the aim of identifying synthetic lethal partner genes for the undruggable gene. Thus, cancer cells carrying the undruggable mutant gene can be selectively killed by specifically targeting the function of the synthetic lethal partner.

Epigenetic regulation is widespread in cancers, involving many targets, and epigenetic-related gene mutations are very common in a variety of cancers [6]. According to epigenetic synthetic lethality approaches, targeting synthetic lethal pair with epigenetic mutations or using epigenetic inhibitors against cancers with “undruggable” mutations may be possible to expand the range of drug treatments and to improve the efficacy of some epigenetic inhibitors in solid tumors.

In this review, we focus on epigenetic-related synthetic lethal interactions in cancers, involving mutations or deletions of epigenetic-related genes and inhibitors of epigenetic enzymes, which might have potential therapeutic value in the future.

## Synthetic lethality between epigenetic alterations and epigenetic inhibitors

### Synthetic lethality induced by ARID1A mutation and EZH2 inhibition

SWI/SNF (SWItch/Sucrose Non-Fermentable), a chromatin remodeling complex mainly including the components ARID1A, SMARCA4, SMARCB1, SMARCD1, and ACTL6A [7], is considered a tumor suppressor, and inactivation of SWI/SNF subunits is thought to drive tumorigenesis by altering gene expression [2, 8, 9]. PRC2 (Polycomb Repressive Complex 2) is another important complex, including EZH2, SUZ12, EED, and YY1, which catalyzes mono-, di-, and tri-methylation on lysine 27 of histone H3 and is involved into the development and progression of multiple cancers [10]. Recently, increasing evidence has shown that there is epigenetic antagonism between the SWI/SNF complex and the PRC2 complex. Therefore, PRC2 complex inhibitors may be potential synthetic lethal treatments for cancers with SWI/SNF mutations or deletions. The gene encoding ARID1A is frequently mutated in multiple cancers. In ovarian clear cell carcinoma, Bitler et al. found that there is a synthetic lethal interaction between *ARID1A* loss-of-function mutation and an inhibitor of EZH2 [11]. They found that GSK126, a specific small molecule inhibitor of EZH2, induced a significant decrease of cell proliferation in *ARID1A* knockdown cell lines. In contrast, the cell proliferation of wild-type *ARID1A* cell lines did not change significantly after treatment with GSK126. Moreover, in the mutant *ARID1A* cell lines, ectopic expression of wild-type *ARID1A* significantly reduced the sensitivity to GSK126. In a nude mouse xenograft model with tumors derived from *ARID1A* mutant cells, they also found that the size of tumors was significantly reduced by GSK126 treatment. The above results demonstrated that the synthetic lethal approach using an EZH2 inhibitor in *ARID1A* mutation patients might be a promising strategy for cancer therapy.

### Synthetic lethality induced by loss of SMARCB1 and inhibition of EZH2/HDAC

*SMARCB1* is widely described as a tumor suppressor gene. SMARCB1, also known as SNF5, is a subunit of the SWI/SNF complex. Complete loss of SMARCB1/SNF5 is very common in malignant rhabdoid tumors (MRT) and atypical teratoid/rhabdoid tumors (ATRT) [12–15]. MRT is a rare but highly malignant cancer with a poor prognosis, and so there is an urgent need for new improved therapies [16]. Experiments have shown that treatment with pharmacological concentrations of EZH2 inhibitors may significantly inhibit the proliferation of SMARCB1/SNF5-deficient MRT cells, while having no significant effect on the proliferation of wild-type SMARCB1/SNF5 MRT cells [17]. Further mechanistic research showed that the expression level of the tumor suppressor gene *p16*^*INK4a*^ is downregulated in SMARCB1/SNF5-deficient tumor cells and increased in EZH2-deficient cells. In SMARCB1/SNF5-deficient tumor cells, EZH2 inhibitor treatment can increase *p16*^*INK4a*^ expression by lowering the level of tri-methylated H3K27 in the *p16*^*INK4a*^ gene region, which demonstrates that EZH2 epigenetically silences the expression of *p16*^*INK4a*^ by catalyzing H3K27 tri-methylation [18, 19]. It was also found that SMARCB1/SNF5-deficient tumor cells are highly sensitive to EZH2 inhibitors, which significantly inhibit cell proliferation and increase apoptosis [20]. In addition, several reports have shown that some HDAC inhibitors can partially mimic the histone acetylation function of SWI/SNF in SMARCB1/SNF5-deficient MRTs, thus indicating that HDAC inhibitors may have anticancer effects in MRTs [21, 22]. Further experiments demonstrated that pan-HDAC inhibitors can significantly inhibit the proliferation and self-renewal ability of SMRCB1/SNF5-deficient MRTs [16]. This study may expand the applications of marketed HDAC inhibitors and uncover potential clinical applications of EZH2 inhibitors.

### Synthetic lethality induced by CREBBP mutation and p300 inhibition

CREB binding protein (CREBBP), also known as CBP, has histone acetyltransferase (HAT) activity [23] and regulates chromatin structure and gene expression. Loss-of-function mutations of the *CREBBP* gene are very common in a variety of cancers. Approximately 10–15% of non-small cell lung cancers (NSCLC) and small cell lung cancers (SCLC) have *CREBBP* loss-of-function mutations [24, 25]. P300/EP300, also known as p300 HAT or E1A-associated protein p300, is a histone acetyltransferase, which regulates the transcription of genes via chromatin remodeling [26]. Screening with an siRNA library revealed that knockdown of the gene *P300*, which encodes the CREBBP paralog P300/EP300, is synthetically lethal with *CREBBP* deletions. The study found that G1-S phase cell-cycle arrest occurs after inhibition of P300 in CREBBP-deficient or CREBBP-knockdown lung cancer cells. The p300-specific inhibitor c646 significantly reduced the growth of CREBBP-deficient cells but not CREBBP wild-type cells. A similar phenomenon has also been found in human hematopoietic cancer [23]. Mechanistically, genome-wide gene expression analysis showed that MYC expression was downregulated in p300-inhibited CREBBP-deficient cells due to decreased levels of acetylation in the promoter region of the *MYC* gene. Expression of the oncogenic transcription factor MYC (c-myc) is upregulated in a variety of cancers. Therefore, it is possible that p300 inhibitors exhibit a synthetic lethal anticancer effect in CREBBP-deficient cancer cells by inhibiting the expression of MYC. This phenomenon provides a potential treatment for CREBBP-deficient cancers.

### Synthetic lethality induced by MLL gene fusion and DOT1L inhibition

Mixed-lineage leukemia (MLL) is a very aggressive form of leukemia which is caused by chromosomal translocations affecting the *MLL* gene. Normally, the *MLL* gene encodes a SET domain histone methyltransferase that catalyzes the methylation of H3K4 [27]. As the rearrangement of the *MLL* gene, the catalytically active SET domain is lost, and *MLL* fuses with other genes such as *AF4*, *AF9*, *AF10*, and *ENL* to generate fusion proteins. The N-terminal portions of these fusion products are lost in the rearrangements, but C-terminal gene-specific recognition elements are retained. These fusion products are capable of interacting with another histone methyltransferase by these recognition elements, such as Disruptor of telomeric silencing 1-like (DOT1L), which is known the unique enzyme to catalyze the mono-, di-, and tri-methylation of histone H3 at lysine 79 (H3K79me1, H3K79me2, H3K79me3) [28–31]. As a result, fusion products gain the ability to recruit DOT1L by retained gene-specific recognition elements. [32–34]. Study shows that leukemia cells with *MLL* fusion genes are hypersensitive to DOT1L inhibitors such as EPZ5676. DOT1L inhibitors induce cell proliferation and apoptosis in *MLL*-rearranged leukemia cells but do not significantly inhibit cell proliferation or apoptosis in non-*MLL*-rearranged leukemia cells. Mechanistic studies have found that MLL fusion products recruit DOT1L which leads to an enhanced H3K79 methylation level in the promoter or enhancer region of MLL-fusion target genes, including HOXA9 and MEIS1, subsequently regulates their expression and mediates dysfunction of cell differentiation and proliferation [35–37]. Thus, treatment with DOT1L inhibitor in MLL fusion cells could reverse the dysfunction. Another mechanism research found that LAMP5 is positively correlated with the MLL fusion protein level and LAMP5 can be activated by H3K79me2 as a direct target which is generated by DOT1L. Therefore, inhibition of DOT1L can abolish the inhibition of autophagy by LAMP5 [38]. It was also found that MLL fusion protein levels decreased significantly after treatment with DOT1L inhibitors. This work may provide a potential therapy for MLL-fusion leukemia [34, 35].

## Synthetic lethality between epigenetic alterations and non-epigenetic inhibitors

### Synthetic lethality induced by SETD2 deficiency and inhibition of WEE1/PI3Kβ-AKT

SET domain containing 2 (SETD2), a histone methyltransferase that is specific for tri-methylation of Histone3 at lysine 36 (H3K36me3), has been shown to act as a tumor suppressor in human cancers [39]. H3K36me3 is frequently lost in multiple cancer types, and this may be caused by loss of the tumor suppressor SETD2 and overexpression of KDM4A (a H3K36me3 demethylase) [40]. WEE1 is a nuclear kinase of the Ser/Thr family and is an important factor in cell-cycle regulation checkpoint and DNA damage checkpoints. WEE1 inhibits CDK1 activity by phosphorylation on Tyr15 and Thr14, and decreased CDK1 activity prevents cells from entering mitosis [41–43]. A role for WEE1 in epigenetic regulation has also been reported. WEE1 can catalyze the phosphorylation of histone H2B tyrosine 37 and regulate histone expression [44, 45]. Pfister et al. and Martinelli et al. found that SETD2-deficient tumors cells such as mast cell leukemia and kidney cancers are very sensitive to WEE1 inhibitors. They found that compared to control cells, *SETD2* knockout cells are hypersensitive to the WEE1 inhibitor Adavosertib (AZD1775) [40, 46]. They further found that H3K36me3 catalyzed by SETD2 promotes RRM2 expression, and WEE1 also promotes RRM2 expression through CDK1; thus, inhibition of WEE1 leads to inhibition of RRM2 [47, 48]. The expression of RRM2, which is a subunit of ribonuclease reductase, is associated with the intracellular level of dNTPs, and decreased expression of RRM2 can result in decreased levels of dNTPs. Inhibition of WEE1 in SETD2-deficient cells consequently results in extremely low dNTP levels, which leads to impaired DNA replication and increased cell death [49]. Therefore, there is a synthetic lethal interaction between SETD2 deficiency and WEE1 inhibition.

Terzo et al. also reported that there is a synthetic lethal interaction between SETD2-deficiency and PI3Kβ-AKT inhibition in kidney cancer [50]. The phosphoinositide 3-kinase (PI3K)-AKT axis is a very important signaling pathway in cell proliferation and it is frequently changed in cancers [51]. PI3Kβ-AKT and its downstream effectors are often overactivated and have high expression levels in kidney cancer [52, 53]. Research shows that kidney cancer cells with *SETD2* knockout or mutation, when treated with PI3Kβ-specific inhibitors, displayed significantly decreased viability and migration compared to cells with wild-type *SETD2*. Treatment of SETD2-deficient or wild-type kidney cancer cells with an AKT-specific inhibitor resulted in similar effects. Therefore, these studies demonstrate that loss of SETD2 is synthetically lethal with PI3Kβ inhibition. They also show that AKT is a key part of the interaction between SETD2-deficiency and the PI3Kβ-AKT axis.

### Synthetic lethality induced by KMT2C mutation and PARP inhibition

Poly (ADP-ribose) polymerase (PARP) is reported to be involved in DNA repair, genomic stability, and programmed cell death [54]. Studies have found that *BRCA1/2* mutations and PARP inhibition are synthetically lethal. The PARP inhibitor olaparib, which was developed according to the principles of synthetic lethality, is the first drug for treating ovarian cancer carrying the *BRCA1/2* mutation. Studies have shown that PARP inhibitors not only have synthetic lethal effects with *BRCA1/2* mutations, but also have synthetic lethal interactions with low-activity KMT2C in bladder cancer. Lysine N-methyltransferase 2C (KMT2C), also known as myeloid/lymphoid or mixed-lineage leukemia protein 3 (MLL3), methylates histone3 at lysine 4 (H3K4), and the *KMT2C* gene has a high mutation rate in bladder cancer [55–57]. Studies have shown that the epigenetic state is changed in bladder cancer cells with low KMT2C activity, and there is decreased expression of genes involved in DNA repair. Therefore, PARP inhibitors have synthetic lethal effects in cancer cells with low KMT2C activity, especially in epithelial carcinoma, such as bladder cancer, colon cancer, NSCLC, and HNSCC [58]. This synthetic lethal interaction may provide a new clinical use of PARP inhibitors.

## Synthetic lethality between non-epigenetic alterations and epigenetic inhibitors

### Synthetic lethality induced by TP53 mutation and EZH2 inhibition

The *TP53* gene, which encodes the widely studied tumor suppressor protein p53, is frequently mutated in various cancers [59, 60]. Previous studies have generally focused on loss-of-function mutations in *TP53*, and have suggested that the tumor suppressor capacity of p53 is lost, thereby promoting tumor development. However, recent investigations have shown that some *TP53* mutations are gain-of-function changes that endow the p53 protein with new activities that can promote tumor development, including increased cell proliferation and cell migration, etc. [60–63]. Zhao et al. used RNA immunoprecipitation sequencing (RIP-seq) to demonstrate that EZH2 binds to the 5′ UTR of *TP53* mRNA, which enhances transcription and translation of p53 protein. This mechanism is independent of the methyltransferase activity of EZH2. Furthermore, it was found that reduction of EZH2 expression by antisense oligonucleotides (ASO) instead of an EZH2 inhibitor induces synthetic lethality in a variety of cancer cells with gain-of-function mutations of p53, such as partial breast cancer and prostate cancer. And there is no significant growth inhibition in wild-type p53 cells [64]. *TP53* mutation is very common in cancers, and therefore the synthetic lethal interaction of p53 and EZH2 may have clinical value for cancer treatments.

## Conclusions

Epigenetic modifications are very common in malignant tumors, and multiple tumor types are dependent on specific epigenetic modifications. Mutations in numerous oncogenes and epigenetic-related genes are also common in a variety of cancers, and many loss-of-function mutations are considered to be undruggable. Therefore, the use of the principle of synthetic lethality is a breakthrough for treating tumors that carry such mutations.

**Fig. 1:**
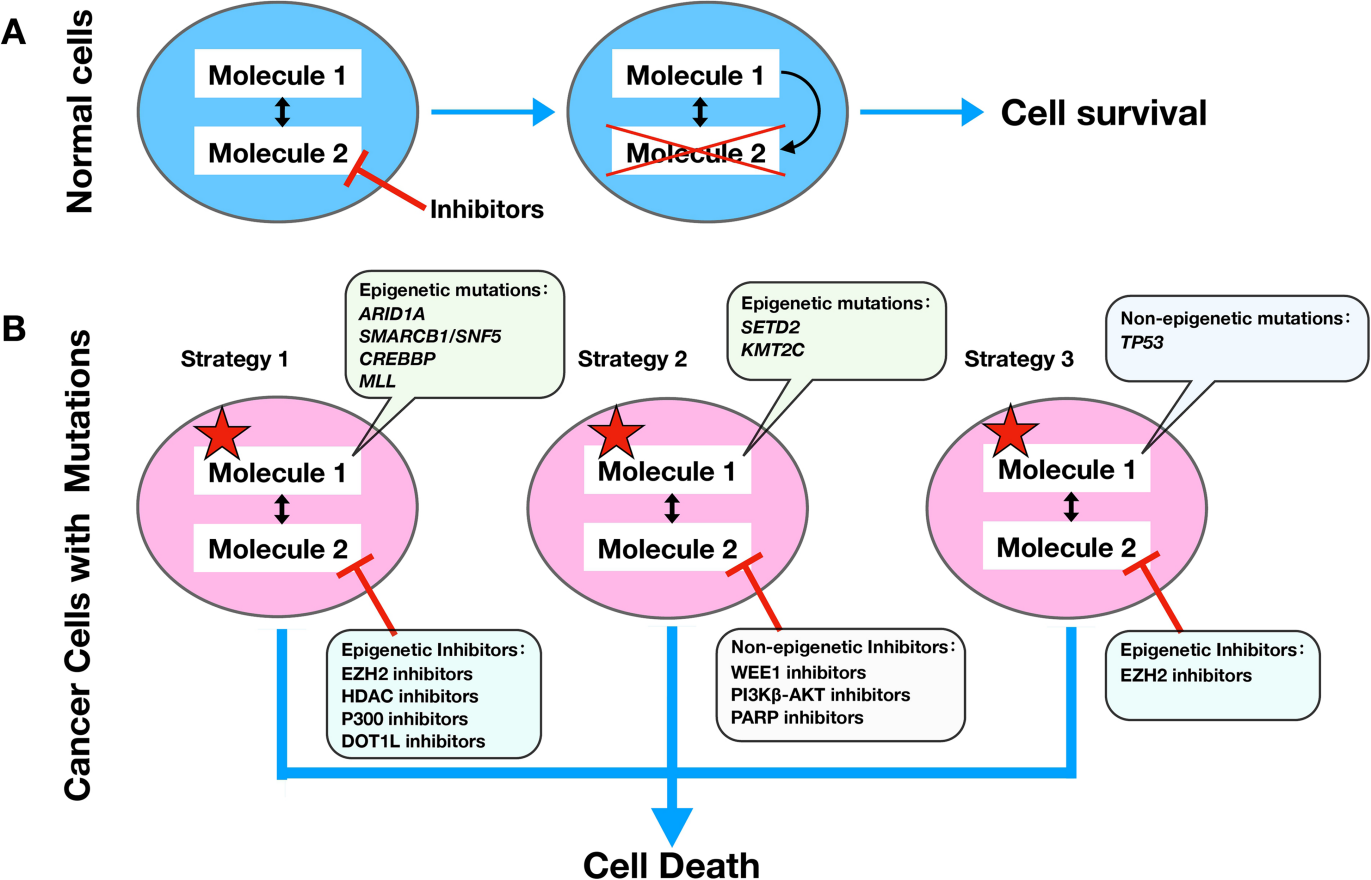
Epigenetic synthetic lethality approaches. **a** In normal cells, inhibiting one molecule in a synthetic lethal pair may lead to a compensatory response of another molecule, and therefore the cells survive. **b** Mutations in cancer cells are represented by red stars. In cancer cells with mutations, one molecule of synthetic lethal part has changed, and the specific inhibitor has inhibited another molecule, so it cannot produce complementarity or antagonism, leading to cell death. This review mainly introduces three epigenetic synthesis of lethal strategies. Strategy 1 uses epigenetic inhibitors in cells with epigenetic mutations cells; strategy 2 uses non-epigenetic inhibitors in cells with epigenetic mutations; and strategy 3 uses epigenetic inhibitors in cells with non-epigenetic mutations. Specific mutations and inhibitors are shown in the figure

Screens using CRISPR and shRNA technology have uncovered multiple sets of epigenetic-related synthetic lethal pairs, such as the SWI/SNF complex and PRC2 complex inhibitors, SETD2 and WEE1 inhibitors or PI3K-AKT inhibitors, KMT2C and PARP inhibitors, MLL fusions and DOT1L inhibitors, p53 and EZH2 inhibitors, and CREBBP and p300 inhibitors. These synthetic lethal pairs and their mechanisms are shown in Table 1 and Fig. 1. The inhibitors are all highly active in tumor cells carrying particular mutant genes but have no significant effect on wild-type cells. Synthetic lethal combinations have also been found that can alleviate the drug resistance of some tumors; for example, AKT inhibitors reverse the resistance to EGFR-TKIs in NSCLC with *PBRM1* mutation, and EGFR-TKIs reverse the resistance to MET and ALK inhibitors in NSCLC with *SMARCE1* deletion [65, 66]. Although research into using synthetic lethality to overcome drug resistance is limited, it will become an important new direction in the fight against drug resistance.

**Table 1:** Epigenetic-related synthetic lethal relationships and mechanisms in different

Gene	Inhibitor	Cancer type	Mechanism	References
Epigenetic alterations with epigenetic inhibitors				
* ARID1A*	EZH2 i	OCCC	Epigenetic antagonism with PRC2	[11]
*SMARCB1*	EZH2 i	MRTs and ATRT	Restores expression of p16^INK4a^	[17, 20]
*SMARCB1*	HDAC i	MRTs and ATRT	Mimics HAT activity	[16]
*CREBBP*	p300 i	Lung Cancer Hematopoietic cancer	Reduces acetylation of MYC promoter	[23]
*MLL*	DOT1L i	Leukemia (MLL)	Reduces H3K79 methylation levels, interfering expression of MLL-fusion target gene inhibit LAMP5 and promote autophagy	[34–38]
Epigenetic alterations with non-epigenetic inhibitors				
*SETD2*	WEE1 i	Kidney cancer mast cell leukemia	Starves the cells of dNTPs	[40, 46]
*SETD2*	PI3Kβ-AKT i	Kidney cancer	Inhibition of the PI3Kβ-AKT axis	[50]
*KMT2C*	PARP i	Bladder cancer Colon cancer NSCLC HNSCC	Blocks HR-mediated DNA repair	[58]
Non-epigenetic alterations with epigenetic inhibitors				
*TP53*	EZH2 i	Breast cancer Prostate cancer	Blocks the binding of EZH2 and p53	[64]

Epigenetic modifications and gene mutations regulate gene expression at different levels. Therefore, the combination of the two ways may make it easier to find synthetic lethal pairs, breaking through the bottlenecks of cancer treatment, especially for some patients who have pathogenic mutations but lack targeted drugs, such as *KRAS* and *TP53* mutations. In addition to screening for synthetic lethal pairs using the CRISPR-Cas9 system and siRNA libraries, screening synthetic lethal pairs for specific gene mutations also can be performed by chemical compound libraries or small molecule inhibitor libraries. And there is often complementarity or antagonism between synthetic lethal pairs, usually in related or same signaling pathway or cell development process. Therefore, it is possible to optimize and narrow the screening range and increase the screening efficiency.

In summary, this review has explored multiple epigenetic synthetic lethal relationships that may provide potential therapies for cancer.

## Data Availability

Not involved into this

## Abbreviations

ALK: Anaplastic lymphoma kinase
ARID1A: AT-Rich Interaction Domain 1A
ASO: Antisense oligonucleotides
ATRT: Atypical teratoid/rhabdoid tumors
BRCA1/2: Breast cancer type 1/2
CDK1: Cyclin-dependent kinase1
CREB1: CAMP Responsive Element Binding Protein 1
CREBBP: CREB Binding Protein
CRISPR: Clustered regularly interspaced short palindromic repeats
dNTP: Deoxyribonucleoside triphosphate
DOT1L: Disruptor of telomeric silencing 1 like
EGFR-TKIs: Epidermal growth factor receptor-tyrosine kinase inhibitors
EZH2: Enhancer of Zeste Homolog 2
HDAC: Histone deacetylase
HNSCC: Head and neck squamous cell carcinoma
KDM4A: Lysine-specific demethylase 4A
KMT2C: Lysine Methyltransferase 2C
LAMP5: Lysosome-associated membrane protein 5
MET: MET proto-oncogene, receptor tyrosine kinase
MLL: Mixed-lineage leukemia
MRTs: Malignant rhabdoid tumors
MYC: MYC proto-oncogene, bHLH transcription factor
NSCLC: Non-small cell lung cancer
PARP: Poly (ADP-ribose) polymerase
PBRM1: Polybromo 1
PI3Kβ-AKT: The phosphoinositide 3-kinase-AKT
PRC2: Polycomb repressive 2
RRM2: Recombinant Human Ribonucleotide Reductase M2
SETD2: SET domain containing 2
SMARCB1: SWI/SNF-related, matrix-associated, actin-dependent regulator of chromatin, subfamily b, member 1
SWI/SNF: SWItch/Sucrose Non-Fermentable
WEE1: Wee1-like protein kinase
